# From the Editor

**DOI:** 10.3390/dermatopathology7010001

**Published:** 2020-06-10

**Authors:** Gürkan Kaya

**Affiliations:** 1 Department of Dermatology, University Hospital of Geneva, 4, rue Gabrielle-Perret-Gentil, 1205 Geneva, Switzerland; gkaya@hcuge.ch; 2 Department of Clinical Pathology, University Hospital of Geneva, 4, rue Gabrielle-Perret-Gentil, 1205 Geneva, Switzerland

Dear Readers,

I have the great pleasure of announcing you that *Dermatopathology*, the open access journal in the field of cutaneous pathology and the official journal of The European Society of Dermatopathology (ESDP), continues under the same title by conserving its PubMed and ESCI indexation with the publisher MDPI, Basel, Switzerland.

The ESDP was first founded in Zurich in 1996 initially with no official character. After my takeover as president of the ESDP in 2011, I have worked for many years for the legalization of the status of the ESDP and the Society was officially founded in Geneva, Switzerland, in 2016. The aims and objectives of the ESDP are to educate by organizing meetings, creating slide sets and providing opportunities for trainees in pathology and dermatology to spend time in centers of dermatopathology of their choice; to enhance co-operation between pathologists and dermatologists interested in dermatopathology; to create a stronger link between dermatopathology and investigative dermatology; to standardize the quality criteria of performance in dermatopathology and to establish a European Diploma of Dermatopathology, which is the equivalent of the Board Examination in the United States, accessible to both pathologists and dermatologists.

For over a century it has been well accepted that in the specialty of dermatology, most major texts and journals have been established and founded primarily from clinical dermatology. In recent years many dermatologists and pathologists with extensive expertise in dermatopathology have been recognized. This journal is an example of this integration. The majority of dermatopathological organizations have a similar proud tradition and the ESDP is no exception. Like the International Society of Dermatopathology (ISDP) and the American Society of Dermatopathology (ASDP) having respectively the American Journal of Dermatopathology and the Journal of Cutaneous Pathology as their official journals, the ESDP also has its official journal, *Dermatopathology*.

It is well known that dermatology and pathology specialties, but especially our patients with skin diseases, profit practically and academically from the recognition and importance of dermatopathology. The foundation of the ESDP undoubtedly paved the way for the first move to develop this medical subspecialty and raise the standards in dermatopathology outside the United States. Moreover, with support from the European Union of Medical Specialists (UEMS) and the ISDP, the ESDP and its founder members have been involved in the International Committee of Dermatopathology (ICDP) Executive Board for training examination and certification.

*Dermatopathology* is a peer-reviewed open access journal which publishes original scientific articles on diagnostic and experimental dermatopathology. The journal was created in 2014 with the Publisher Karger based in Basel, Switzerland, as an online journal, and indexed by PubMed and Emerging Sources Citation Index (ESCI) in 2015 and by the Directory of Open Access Journals (DOAJ) in 2016. Case reports, clinico-pathological correlations, controversial issues, basic, translational or clinical research studies related to cutaneous biology and skin disease and studies based on current molecular technologies are welcome. The aim of the journal is to share interesting and most recent clinico-pathological information with the community of dermatopathology all over the world and to create a stronger link between dermatopathology, clinical dermatology and investigative dermatology. *Dermatopathology* also provides a resource for continuing medical education in cutaneous pathology. Currently, the journal publishes original articles, research articles, review articles and case reports in the following sections: “Clinico-Pathological Correlation in Dermatopathology” and “Molecular Dermatopathology”. Starting from this issue (volume 7, issue 1), three novel sections will be included: “Artificial Intelligence in Dermatopathology”, “Pediatric Dermatopathology” and “Experimental Dermatopathology”. After peer-review and final acceptance, articles will be published online very quickly, and therefore be present in the PubMed, ensuring the rapid availability of scientific knowledge for all clinicians and researchers interested in this subspecialty. According to the open access principle, the contents of the journal can be retrieved at all times with no charge and the copyright remains with the authors. I believe that this journal will be a good complimentary addition to the other existing journals in the field and will help to increase the quality of dermatopathology. I invite all the members of the ESDP and all dermatologists and pathologists interested in dermatopathology to submit original articles to *Dermatopathology*.

I would like to thank all the Editorial Board members and the Associate Editors for their tremendous work and help during the past 6 years since the first launch of the journal in 2014 and I also would like to thank them and the new members of the Editorial Board for their kindness to accept to be with me in the second phase of this journey with the new publisher MDPI. As you can see, the old and new members of the editorial team of this journal are, among others, the world’s leading names in dermatopathology. I am sure that their precious contribution will make this journal one of the best journals in the field. I also would like to sincerely thank the team of MDPI who helped me to continue with this project.

As I wrote in my Editorial of the first issue in 2014:

Non quia difficilia sunt non audemus, sed quia non audemus, difficilia sunt.*

* It is not because things are difficult that we do not dare, but because we do not dare, things are difficult (Seneca, Letter to Lucilius, letter 104, section 26, line 5).

Now it is time to pick up the pen!

Sincerely yours,
Gürkan Kaya, M.D.-Ph.D.
Editor-in-Chief
Dermatopathology
President

European Society of Dermatopathology

